# Coordination Modes and Different Hapticities for Fullerene Organometallic Complexes

**DOI:** 10.3390/molecules17067151

**Published:** 2012-06-12

**Authors:** Delia Soto, Roberto Salcedo

**Affiliations:** Institute of Materials Research, National Autonomous University of Mexico, External Circuit, University City, Coyoacan 04510, Mexico City, Mexico

**Keywords:** fullerene, exo organometallic complexes, hapticities

## Abstract

The different coordination modes in fullerene organometallic complexes are reviewed. The main modes are η2 and η5, but there are some interesting studies about the other four, all of them are revised in order to show which is the state of art of this kind of compounds with the respect of the hapticity.

## 1. Introduction

Studies of the organometallic and coordination chemistry of fullerenes started [1,2] immediately after the first reported preparation [3] of these wonderful molecules [4]. Fullerenes have extraordinary capabilities to coordinate metal atoms, both inside (endohedral) and outside (exohedral) the carbon cage. This review will focus on the several kinds of exohedral metallofullerenes in which the metal atom can coordinate with a specified number (n) of carbon atoms in the surface of the cage (hapticity). There have been several good reviews examining various aspects of exohedral and endohedral organometallic complexes of fullerenes [5,6,7]. Good reviews also exist which discuss the exo-organometallic complexes of fullerenes [8,9]. A number of different features found in these species are considered, however one important topic related to these molecules concerns variations in hapticity and their consequences; this topic has not yet been reviewed and will be the subject of our study.

## 2. The Possibilities of Hapticity

**Figure 1:**
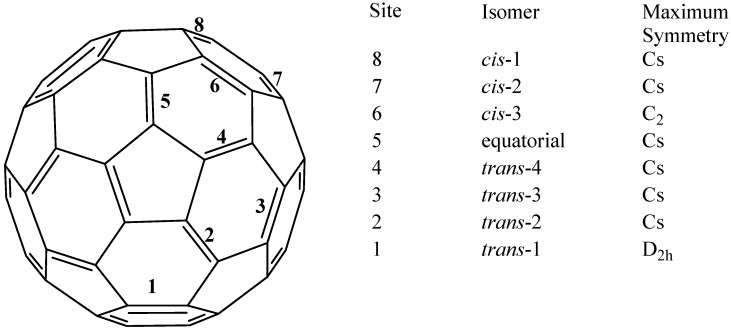
Regiochemistry of double addition to fullerene C_60_ [10].

An interesting characteristic of fullerenes is that in spite of their marked symmetry, they have several different coordination positions. Fullerene can be doubly substituted [in (6,6) fashion] giving regioisomers that have been studied and for which Hirsh has proposed a nomenclature [10]. Taking this nomenclature as a starting point, it is relatively easy to distinguish a point of reaction and possible isomers (see Figure 1); we can have either three *cis* isomers with addenda on the same hemisphere, or an equatorial isomer and four *trans* isomers with addenda on the opposite hemisphere.

**Figure 2:**
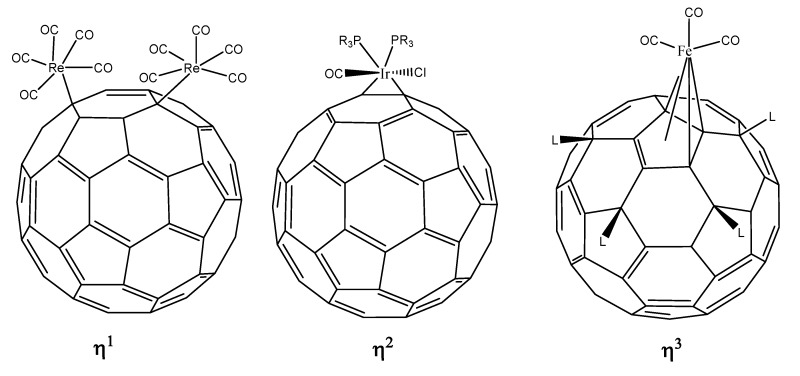

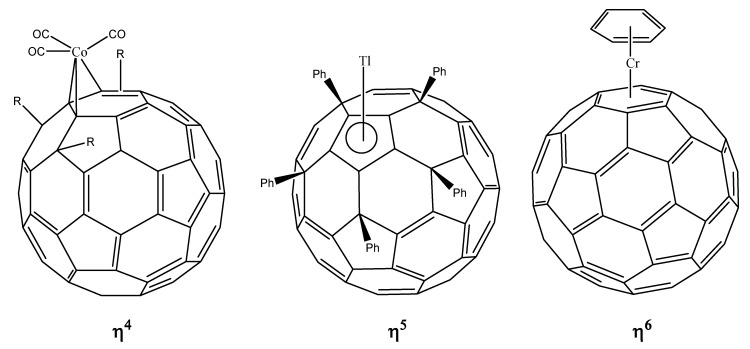
Presenting all possible hapticities for fullerene C_60_.

Lichtenberger [11,12] and his co-workers carried out very interesting theoretical studies on this topic, considering all possible interactions between fullerene C_60_ and a palladium atom. Similarly Sokolov [13] suggested a very useful classification of the coordination modes. All these ideas have been resumed in the following list and in Figure 2.

﹣ η^1^ hapticity with the metal atom directly above any carbon atom (an σ bond is expected);
﹣ η^2^ hapticity; this may be (6,6) or (6,5) type, although the last of these is not common;
﹣ η^3^ hapticity; the metal atom linked to three carbon atoms, theoretically this could be on either a six or five-member ring;
﹣ η^4^ hapticity, the metal atom linked to four carbon atoms;
﹣ η^5^ hapticity, metal atom linked directly above the center of a five-member ring;
﹣ η^6^ hapticity, metal atom linked directly above the center of a six-member ring.

Theoretically it is possible to produce examples of all six items; however in practice there are strong limitations, therefore each of these cases will be presented in turn.

## 3. η^1^ Hapticity

This coordination mode is a little ambiguous because it represents the only example where the bond is σ, however there are certain small examples where there is only one bond (σ) between a fullerene and a metal atom [14,15,16]; likewise there are some cases where a complex can manifest a very complicated coordination, but some of the bonds present a possible σ coordination [17]. Besides this, a number of interesting theoretical studies exist, discussing this aspect as well as other features meriting comment [18].

**Figure 3:**
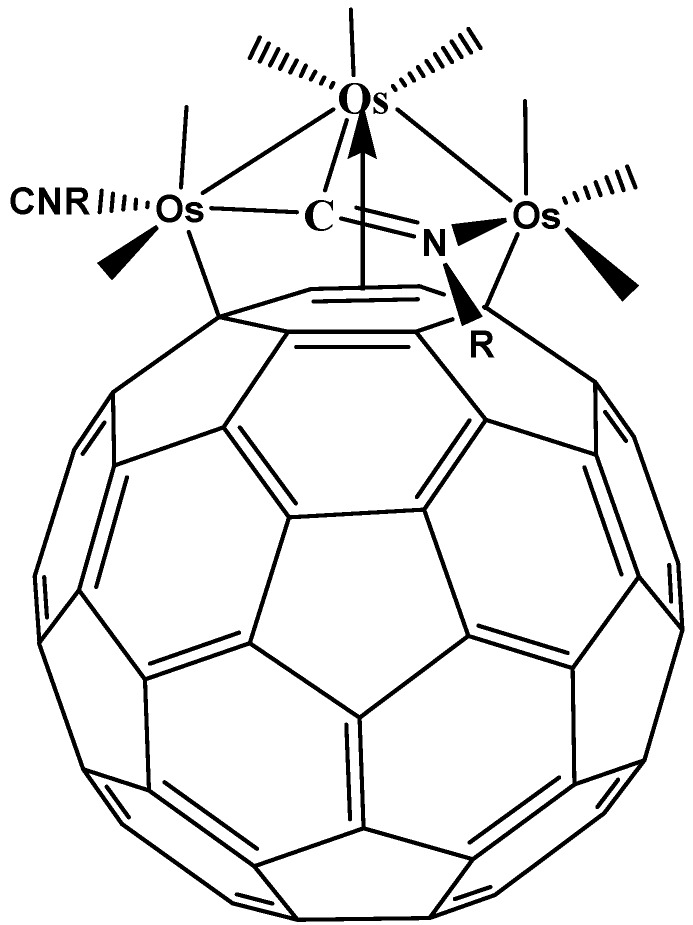
Model of a compound with η^1^ hapticity occurring in a single bond [20].

The salient point referring to this type of coordination is the nature of the bond σ (as this represents the only type of compound with this type of bond) or π. There have been interesting discussions about “hapticity” involving only one carbon atom, for example the possible accommodation found in C_60_{Ag(NO_3_)}_5_ [19] or the clusters of triosmium in the complexes prepared by Park [20] (see Figure 3). Indeed this coordination seems to be neither stable, nor strong. There are also some interesting ionic derivatives [21,22] for which the presence of the σ bond and its relevance have been described and these have been most elegantly studied by applying x-ray crystallography and theoretical calculations. In this case, the authors find that the bond between fullerene and the metal atom (cobalt) is larger than usual and in some way stabilized by the crystal lattice.

There are some cases where the defined structure is not clear and therefore the probability of a structure which includes a σ bond has been suggested, although there are other possible explanations [23]. Cases like this require a lot of work in order to confirm the existence of this particular coordination.

Theoretical studies referring to this ubiquitous coordination are not common; the η^1^ coordination has been considered more as a possible step in a reaction or fluxional process [11,12] than as a defined structure. Almost all the available information referring to this topic has been discussed here, however it is important to note the interesting theoretical analysis carried out by Park and his group [18] where the electronic population of the bonds involved in the interaction of the metal plays a fundamental role in explaining hapticities η^1^, η^2^ (6,5) and η^2^ (6,6). They claim that η^2^ (6,6) should represent the most stable structure out of the three in the case of neutral molecules, but the ionization of these as a result of successive electron reduction produces a stable η^1^ (σ bond) species [24], therefore this may be a good strategy for preparing compounds of this kind.

## 4. η^2^ Hapticity

The case of η^2^ is very different from the first example and many reports deal with this coordination mode [5,13]. The main outstanding factor is that practically all the organometallic complexes of this kind have been described as forming the bond in a (6,6) fashion, *i.e.*, the π bonds are always formed in the junction of two six-member rings at the fullerene surface. The hapticity example pertaining to the (6,5) type previously mentioned in the Park study [18] may be important and will be analyzed at the end of this section.

Fullerene can be doubly substituted [in (6,6) fashion] giving eight regioisomers as previously mentioned [10]. A short time after the discovery of C_60_ (and other fullerenes), the organometallic chemistry of these compounds started with the first reports describing these kinds of substance, with the possibility of producing stable η^2^ derivatives [1,25,26]. However work in this area is still ongoing [27,28].

**Figure 4:**
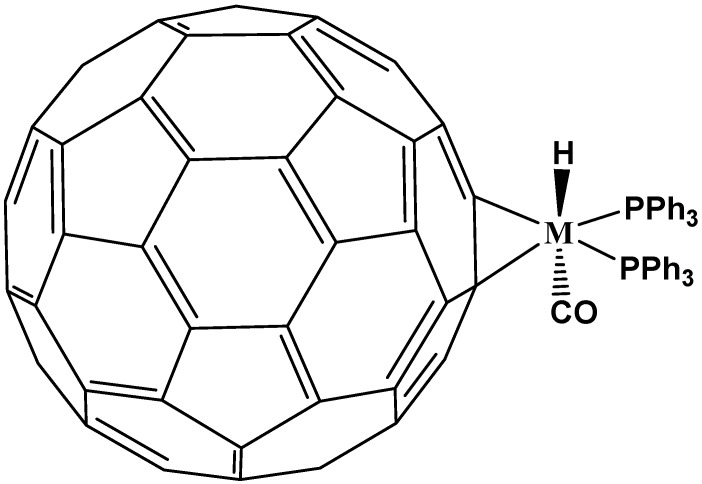
Typical η^2^ fullerene organometallic complex [5].

The great majority of fullerene organometallic compounds with an undisturbed electronic system involve only η^2^ coordination (Figure 4). Thus, it has been suggested [16] that the chemistry of this kind of compound might be similar to that found in the isolated olefinic double bond, where the non-planarity of the fullerene surface compels the orbitals outside this plane not to be completely parallel and therefore the η^5^ and η^6^ coordination manifest bonding problems with a predictable resultant distortion in the formation of the sphere. This feature is a consequence of the limited π conjugation of the fullerene species and it has been suggested [29] that the source of this limitation is precisely its curvature which causes an energetically unfavorable arrangement for the double bonds. Indeed this was also the reason for the predominance of the η^2^ coordination, where there is a lower demand on π orbitals, than on the other hapticities.

An excellent theoretical work provides an accurate description of the features found in this kind of compound. This article by Sgamellotti and his group [30] provides an analysis based on the Dewar-Chatt-Duncanson model (DCD) that was developed in order to explain the nature of the bond between the double bond of olefins and metal atoms, therefore it should be useful also to explain the bond in the fullerene η^2^ complexes. The DCD model propose two bond components, the first one is the donor acceptor interaction (σ type) whereas the second one the dative component (π type), both have important contribution, the first is the responsible of the electronic transfer from the olefin to the metal and is described as the interaction between an empty orbital of the metal and the filled π orbitals of the ligand. The second one involves the back-bond in reverse sense that arises from the interaction between an occupied d-orbital of the metal and a nonbonding π* molecular orbital of the olefin, this last one interaction should be particularly important in the case of fullerene complexes because this last kind of orbitals has large population and is very near the occupied orbitals, therefore the interaction can be strong [31,32]. The authors elegantly demonstrate that the bond makes several contributions and the π back-donation representing the dominant one of these, turns out to be even more important than the direct σ donation. Another interesting point here is that the (6,6) double lost its intrinsic double character, demonstrating a length increase that follows the Pd < Ni < Pt order, as well as corresponding bond energy terms and the concomitant C_60_ distortion. The same bond energy terms are higher for fullerene than the corresponding ethylene complexes, when an analogue π bond is present (together with unsubstituted ethylene).

Other theoretical works have focused on the stability of all the different possible coordination modes. The works of Lichtenberger and his group [11,12] have been mentioned previously, where they found that the only possible stable coordinations for unperturbed fullerene (C_60_) are η^2^ and η^5^, with the former being the most probable, and with all other cases (with the particular exception of the σ η^1^ cases) being repulsive. Likewise in his study, Loboda [33] presents similar results to those of Lichtenberg, but furthermore he suggests that the strongest M-C_60_ bond will be that pertaining to the complex manifesting a large HOMO-LUMO gap, so that the elements in the fourth and fifth series of the periodic table will demonstrate better bonds than those in the third row; referring in all cases to η^2^ hapticity.

**Figure 5:**
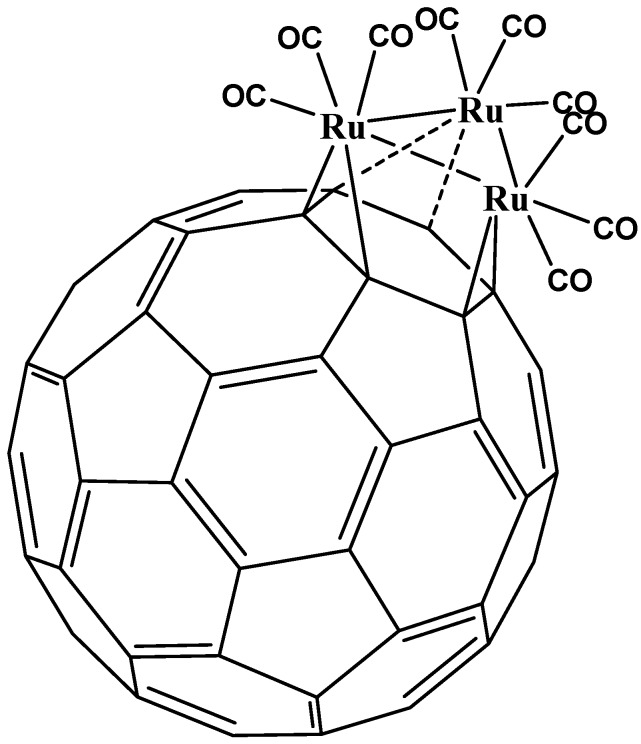
A complex with several η^2^ substitutions in one face [5].

**Figure 6:**
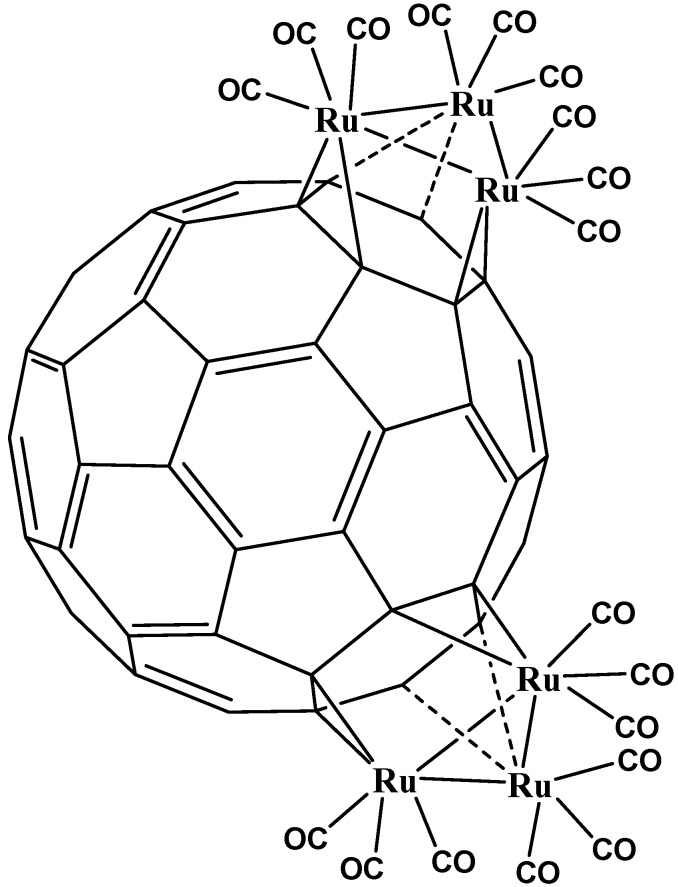
A complex with several η^2^ substitutions in different faces [5].

The addition of fragments containing metal atoms in η^2^ fashion does not greatly affect the electronic distribution on the entire sphere of fullerene, especially in zones far from the coordination and neither does its intrinsic electron affinity change; so that it is possible to envisage multiple substitution taking place at the surface of the sphere or even on the same hexagon. It is also possible to find compounds where the coordination mode is η^2^ but they support multiple substitutions on the same face of the sphere [34] (see Figure 5) or several substitutions on different faces [35] (see Figure 6). However all these kinds of compounds belong to the same coordination mode, therefore the η^2^ has ample versatility in terms of design or for the creation of new substances.

The (6,6) coordination mode for this particular hapticity is a consequence of electron richness because the “best” double bonds for an olefinic coordinative substitution should be those corresponding to the six-member rings, as well as those implicated in weak aromatic systems *i.e.*, the hexagon-hexagon fragments. This is because it has been suggested that six-member faces constitute weak aromatics, whereas five-member faces are anti-aromatic [36].

With respect to the η^2^ (6,5) mode, Park and his group have made interesting theoretical propositions in the same paper mainly focusing on the η^1^ mode [18], arriving at the conclusion that in multiple substitutions and ionic species, the η^2^ (6,5) mode may be more stable than its (6,6) counterpart. This phenomenon may be a consequence of both steric effects and a change in the electronic distribution of the ions.

## 5. η^3^ and η^4^ Hapticities

The formation of η^3^ and η^4^ complexes of fullerene with transition metals does not produce stable systems because the fullerene has an extended conjugated system and the fragments have low polarizability, involving three or four atoms interacting with metals. Thus, the way to form complexes with hapticities 3 and 4 is to modify the fullerene structure by either adding radicals to certain carbon atoms or by replacing certain carbon atoms with hetero-atoms. These are able to change the local structure of the frontier orbitals causing substantial stabilization to complexes, as a result of the transition metal atoms becoming coordinated to three or more carbon atoms. The most pronounced effect occurs when the conjugated system can be divided into two or more independent fragments [37].

**Figure 7:**
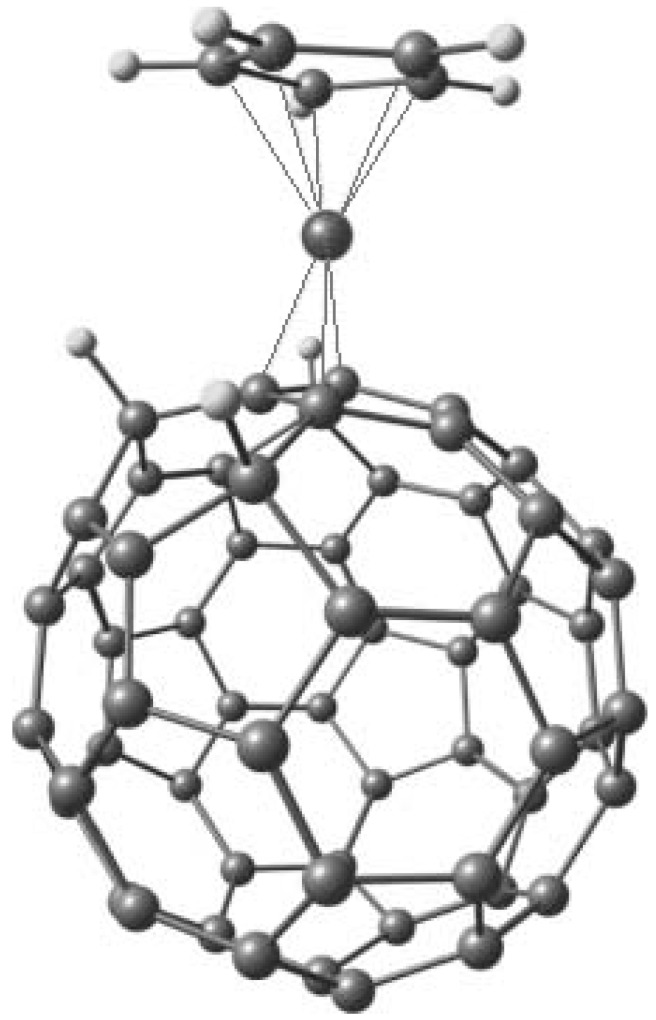
η^3^ hapticity model [38].

Very few articles discuss η^3^; however Stankevich [38] has provided a theoretical work. This article proposes the possible existence of η^3^-complexes between allyl type derivatives of C_60_ fullerene (C_60_R_3_ (R = H, F, Cl, Br)) and Ni or Co, identified by means of DFT_PBE calculations. The study demonstrates that C_60_R_3_ ligands can form the stable species C_60_R_3_Co(CO)_3_ and C_60_H_3_NiC_5_H_5_, where the metal atoms are π-bonded to the fullerene carbon cage with η^3^ hapticity (see Figure 7).

Rubin and his co-workers [39] have presented theoretical and experimental data referring to the existence of complexes comprising a pentaarylfulleride anion and AuPPh_3_ and AgPPh_3_. In conformity with the distances and angles of bonds between the metal and the cyclopentadienyl ring embedded in the fullerene, they observed several coordination modes ranging from η^1^ with a slight distortion toward η^3^ in the case of gold, and to η^2^/η^3^ in the case of silver. 

**Figure 8:**
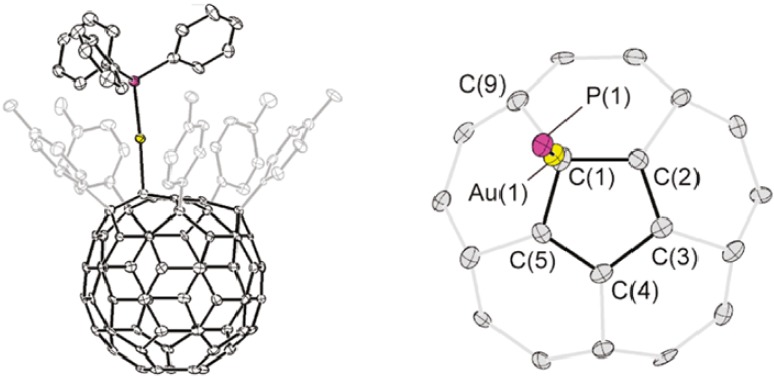
Rubin’s proposition for a η^1^ to η^3^ hapticity change in the case of a gold complex (Reproduced from [39] with permission).

**Figure 9:**
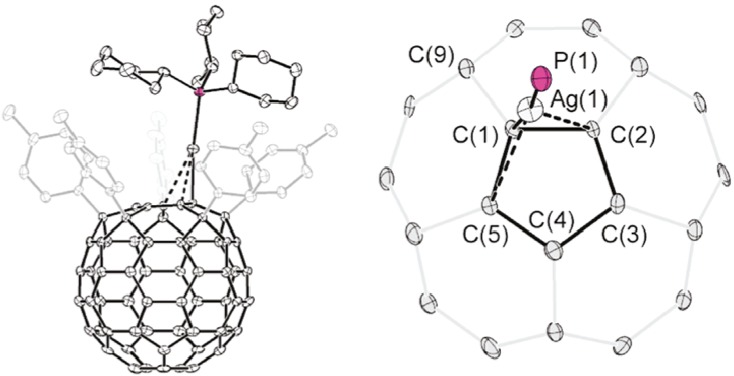
Rubin’s proposition of η^3^ hapticity for a silver complex (Reproduced from [39] with permission).

The embedded fullerenyl cyclopentadienyl ring (Figure 8) displays a slightly distorted η^1^ coordination to the Au(PPh_3_) fragment leaning toward η^3^ (denoted η^1^/η^3^), with a principal Au(1)-C(1) distance of 2.160(6) Å and secondary Au(1)-C(2) and Au(1)-C(5) distances of 2.823(7) and 2.782(5) Å. In the case of silver (Figure 9), the principal Ag(1)-C(1) distance of 2.259(4) Å and the secondary interactions Ag(1)-C(2) and Ag(1)-C(5) with distances of 2.525(4) Å 2.894(4) Å, together with a reduction in the metal-ligand bond angles Ag(1)-C(1)-C(2) (82.7(2)°) and Ag(1)-C(1)-C(5) (100.8(2)°), and likewise a corresponding increase in the angle Ag(1)-C(1)-C(9) (113.8(3)°) are all factors pointing to an increase in the hapticity of metal-ligand bonding from η^1^ to η^3^.

The possibility of the formation of η^4^-π-complexes of C_60_ with a Fe(CO)_3_ species was theoretically analyzed by Chistyakov and Stankevich, applying the DFT_PBE approach [40]. It was found that the attachment of four (or six) hydrogen atoms to C_60_ to form “butadiene”-or “fulvalene”-type derivatives of C_60_ fullerene, respectively, promotes the stabilization of η^4^-π-complexes of C_60_ derivatives with Fe(CO)_3_ unit, but even when the energies calculated are favorable, the comparison with the energies of η^5^-π-bonds in ferrocene molecule (110 kcal mol^−1^) and η^5^-π-C_60_H_5_FeCp (117 kcal mol^−1^)and η^5^-π-C_40_H_5_FeCp (116 kcal mol^−1^) complexes shows the η^4^-π-bonds to be weaker than the η^5^-π-bonds.

## 6. η^5^ Hapticity

The formation of stable η^5^-complexes between fullerenes and transition metals is highly improbable because the conjugated system is rather strongly delocalized, and the polarization of the atoms of the five-member face is weak. Lichtenberger [11,12], Sokolov [13], Loboda [33], Rogers [41] and Kang [42] have carried out theoretical analyses on this particular coordination mode. There is a suggestion that in order to disrupt the conjugated system, it is necessary to modify the fullerene structure by the addition of R groups, hydrogen (H), methyl (Me) or phenyl (Ph), in the α-position with respect to the same five-membered face. Chistyakov and Stankevich [43] in particular have suggested that the formation of the cyclopentadienyl ion in the fullerene is the key to forming complexes with transitional metals. Thus there are two challenges; one is to form a fence around the five-member ring and the other to impose the necessary ionization in order to produce the cyclopentadienyl group.

**Figure 10:**
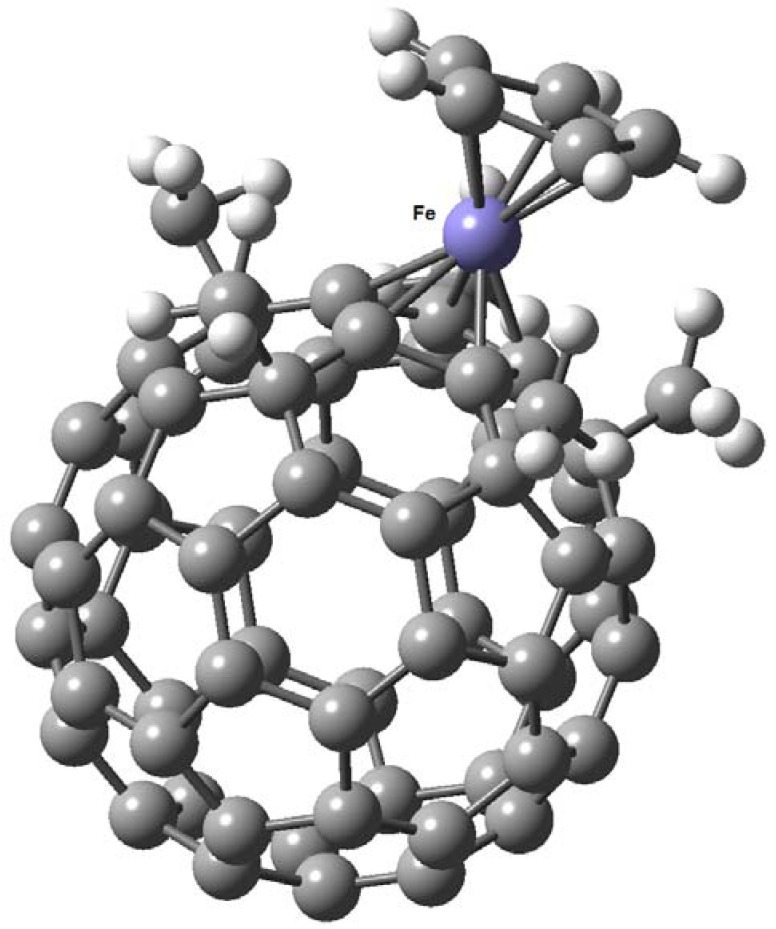
Molecular structure of ferrocene/C_60_-fullerene hybrid determined by X-ray diffraction (reproduced from [46] with permission).

Nakamura and his coworkers accomplished this task [44]; they built a “fence” of five organic groups around the five-member ring and obtained the corresponding compounds. Practically all works in this field are derived from this group, for example they published [45] the synthesis of the first complexes called (pentamethylfullereno)/ferrocene which were synthesized in two steps from C_60_-fullerene with a 45% overall yield, based on their previous work with cyclopentadienyl (C_60_R_5_, R=Me) and indenyl (C_70_R_3_, R=Me), both metal complexes of fullerenes [46]. The distances between the pentagon carbon atoms and iron (2.033 Å for Cp-Fe, and 2.089 Å for FCp-Fe) are comparable to those in known ferrocene derivatives (see Figure 10).

**Figure 11:**
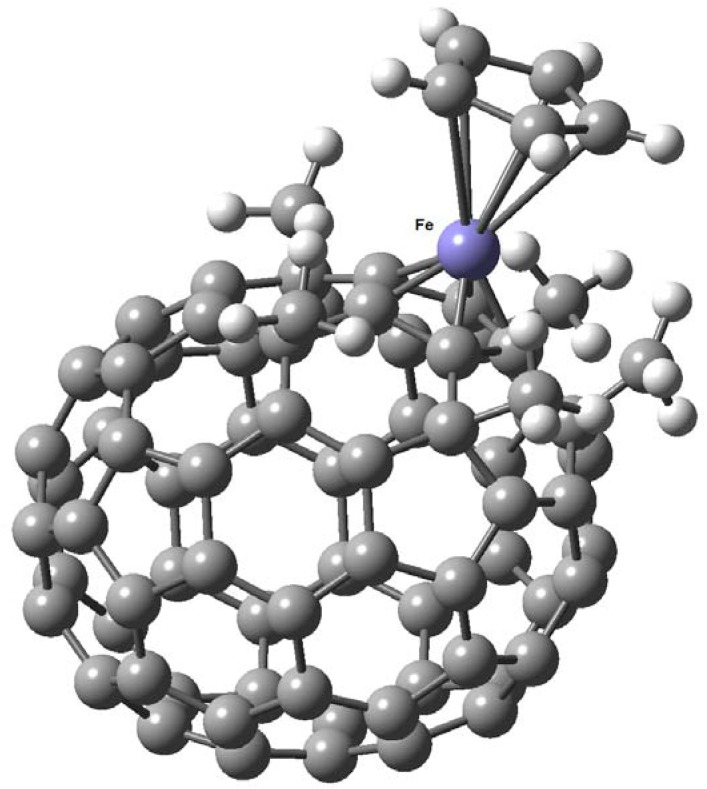
Molecular structure of ferrocene/C_70_-fullerene hybrid, determined by X-ray diffraction (Reproduced from [47] with permission).

**Figure 12:**
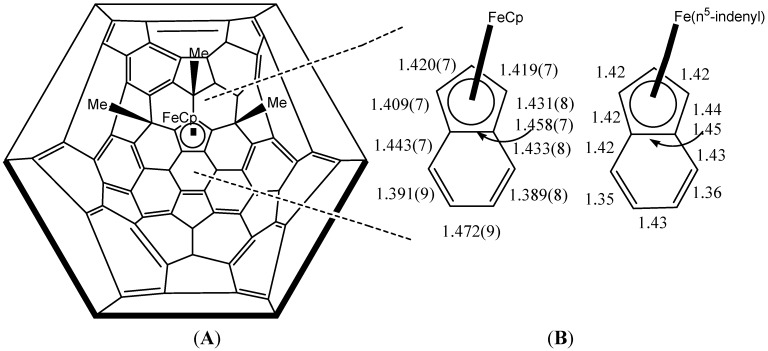
Indenyl iron(II) structure identified in ferrocene/C_70_-fullerene hybrid (**A**) compared to that from Fe(η^5^-indenyl)_2_ (**B**). Bond lengths are in angstroms deviation (Reproduced from [47] with permission).

In another work, Nakamura [47] describes the existence of a ferrocene/C_70_-fullerene hybrid; in this case only three peripheral organic groups are sufficient to form the complex (Figure 11). The structural features of the ferrocene moiety with C_70_ are similar to those with C_60_. With a small but significant difference between ferrocene/C_60_-fullerene and ferrocene/C_70_-fullereneis that the cyclopentadienide C–C bond (1.458 Å) connected to a hexagon in the “belt region” of C_70_-fullerene core is slightly longer than the remaining four C-C bonds in the pentagon, a known feature in the case of indenyl iron complexes. Bond alternation found in the six-member ring next to the cyclopentadienide moiety is another characteristic pertaining to indenyl complexes (see Figure 12).

Further analysis of this hybrid system made clear the unusual stability of the penta-adduct in water, which serves as a precursor to the pentahaptofullerene metal complexes M(η^5^-C_60_R_5_). These compounds represent a rare example of hydrocarbon anions with well-defined water solubility [44]. Consequently, it is not only expected that diverse biological activities will be identified, but also that it will be feasible to create ordered aggregate structures that can be utilized in material science.

Other successful examples of these systems include: (a) the synthesis of complex Rh(η^5^-C_60_R_5_)(CO)_2_, [47], where the average value of Rh-C(Cp of fullerene) distances are shorter when R=Me, than when R=Ph, 2.26 Å and 2.29 Å, respectively; (b) the synthesis of rhenium-hydrofullerene complexes, (CO)_3_-Re-η^5^-C_60_(H)_3_(PhCH_2_)_2_(Ph) [48] and the modification of R-groups in M-η^5^-C_60_R_5_ (M=Re, Fe) by different alkil groups in order to identify new applications in biology, chemistry and other material sciences [49]; (c). The complex with ruthenium(II) where starting from Ru(η^5^-C_60_R_5_)Cl(CO)_2_, the carbonyl ligands can be cleanly replaced in order to obtain the phosphine and isonitrile complexes, without causing cleavage of the fullerene-metal bond [50]. In this work the authors propose that synthesized Ru-complexes can be applied to catalytic reactions both under neutral and reductive conditions; (d). For complexes with Ir (I) [51], Ir(η^5^-C_60_Me_5_)(CO)_2_ which was obtained as starter material stands out. According to electrochemical analysis, this complex is very robust even under harsh reduction. X-ray diffraction study has revealed that Ir-C distance is slightly longer than that of the Cp analogue (2.30 Å *vs.* 2.26 Å). The Ir(η^5^-C_60_Me_5_)(CO)_2_ complex, and unlike its analogue in η^2^ is quite stable toward molecular oxygen, water and heat (100 °C) both as a solution and also as a solid, therefore representing an improved system for future applications.

**Figure 13:**
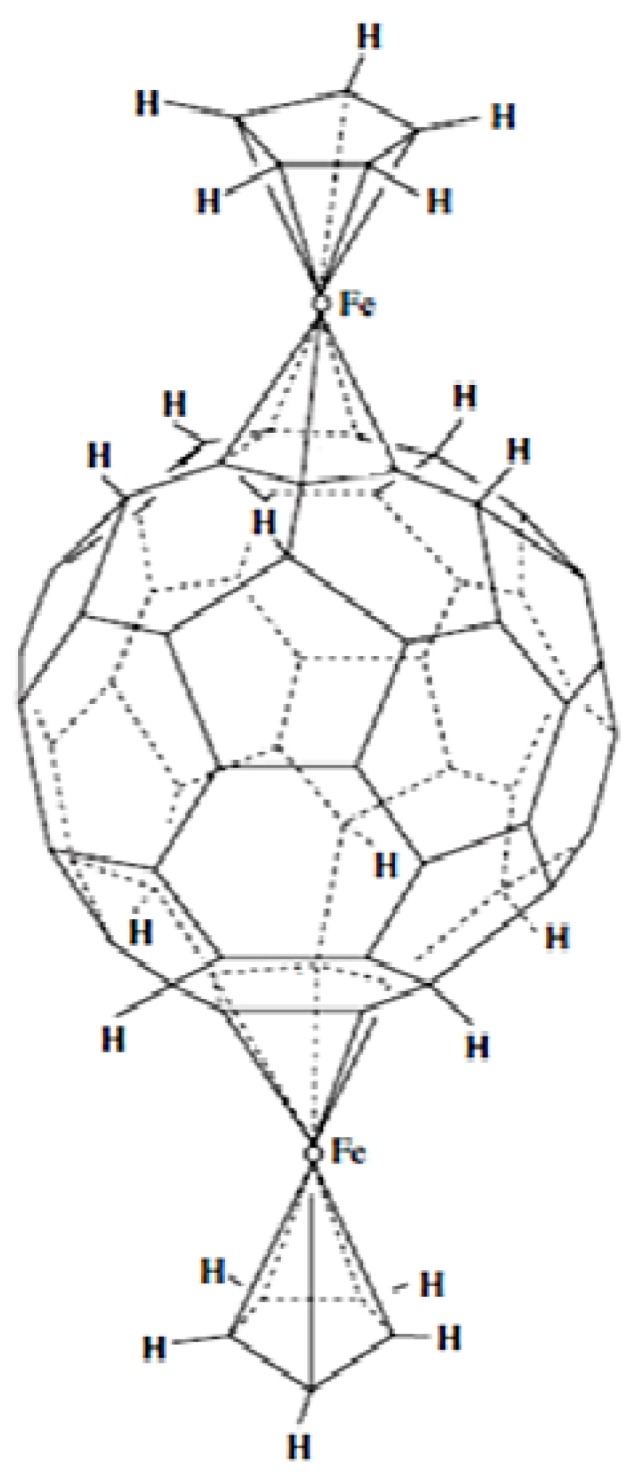
Complex 2η^5^-π-(CpFe)-C_60_H_10_ [51].

A relationship between η^5^ and η^4^ and η^3^ has been suggested by Nakamura in some of the works cited here; indeed Chistyakov and his co-workers theoretically follow this possible transformation [52]. They have studied the molecular and electronic structures of the (CpFe)-C_60_H_10_-(FeCp) complex of the *D*_5*d*_ symmetry, where Cp is the cyclopentadienyl radical, by applying the *ab initio* Hartree-Fock-Roothaan method in the 3-21 *G* basis set. Each FeCp semi-sandwich moiety is linked to atoms of one five-member ring, arranged on the opposite faces of fullerene, by the η^5^-π-type bond (Figure 13). It is apparent that the energy of the η^5^-π-Fe–C_60_H_10_ bonds in the (CpFe)_2_C_60_H_10_ complex is comparable to that of the η^5^-π-Fe–Cp bond in the FeCp_2_ ferrocene molecule (~78 kcal/mol). This system is of particular interest for the formation of quasi-linear polymers with conducting properties.

This same idea, *i.e.*, that of double-decker ferrocene complexes was also studied by the Nakamura group [53,54]. They carried out works following both theoretical and experimental arguments and found that the addition of a second ferrocene fragment caused strong instability and may generate very short-lived radical-ion pairs.

This particular coordination mode has been particularly studied (along with η^2^) because of the large number of compounds created by Nakamura. Importantly, although Nakamura and his colleagues [55] published some articles about his work, however it appears that certain areas have been left for other research groups to develop [56]. 

## 7. η^6^ Hapticity

Several authors have mentioned the particular limitation of fullerenes, when attempting to form η^6^ complexes. Lichtenberg and his co-workers [11,12] presented the results of quantum chemical calculations where they identify attractive interaction for metal atoms only in the cases of η^2^ and η^5^ coordination modes, with the former being predominant. 

Chistyakov and his group [40] compared η^5^ and η^6^ cases again using theoretical methods and also concluded that η^6^ is not a good example. However Stankevich and his group [43,57] indicate that a perturbation of the system, consistent with the addition of R groups to the surface of the C_60_ in six α positions with respect to a common six-member ring is able to stabilize the coordination of a metal atom in η^6^ manner. A similar calculation was performed by Rogers and Marynick [41] considering an C_60_Cr(CO)_3_ complex, where they concluded that the binding energy is lower than in the case of an analogous compound containing only a benzene ring instead of the fullerene, but they concluded that the complex should be stable.

In the experimental context, two reports have cited possible η^6^ complexes. Firstly, Peng and his group [34] prepared C_60_RuCp(Me)_5_ and by analyzing mass spectroscopy data they proposed that the coordination in this compound is η^6^. Secondly Taylor and his co-workers [58] have prepared a molybdenum organometallic derivative of C_60_ with the polysubstitution of fluorine atoms and suggested that this coordination is also η^6^, although the C_60_ sphere manifests a strong distortion. 

**Figure 14:**
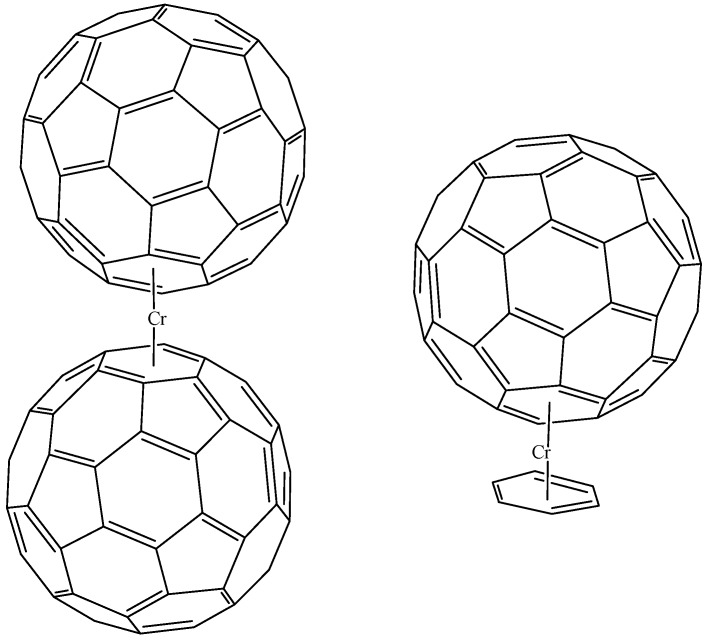
Hybrid compounds suggested by Salcedo [59].

Salcedo carried out simulations on two possible kinds of compounds (see Figure 14). In the first instance, he proposed the possibility of an analogy of dibenzen-chromium [59] but with C_60_ fullerenes present, instead of the normal benzene rings. His calculations indicate a very stable species. In the second instance, a mixed complex, where there is a C_60_ on one side and a benzene ring on the other is also analyzed. It is of note that both molecules demonstrate interesting electronic behavior and can be considered to represent semiconductor species.

**Figure 15:**
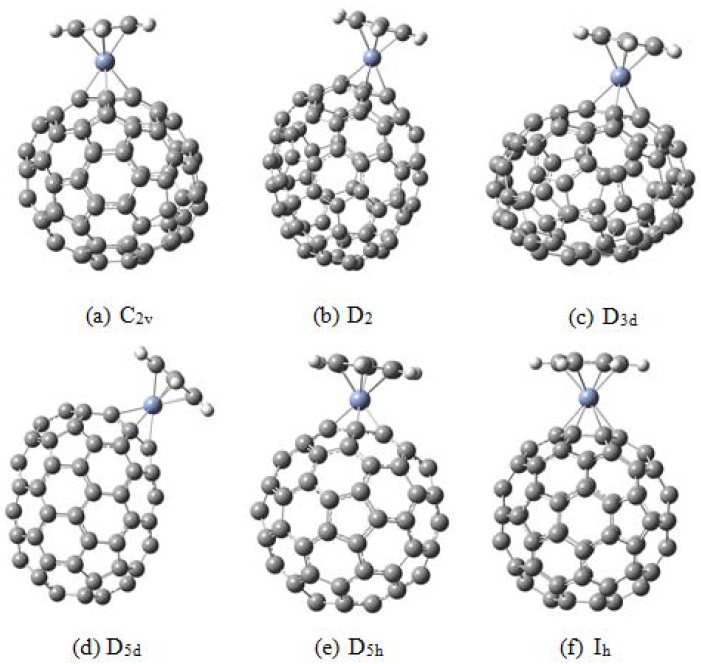
The six isomers of C_80_CrC_6_H_6_ [61].

A possible problem for the η^6^ coordination would appear to be the curvature of the fullerenes [13,60]. One way to avoid this situation would be to prepare organometallic compounds of fullerenes larger than C_60_ and C_70_. This topic was discussed in the article by Molina, Pérez-Manríquez and Salcedo [61] where they carried out an interesting comparison of all possible isomeric complexes with the formula C_80_CrC_6_H_6_ (Figure 15). The results show that compounds derived from C_2v_, D_2_, D_3_ and D_5d_ isomers of C_80_ will comprise η^6^ stable species with interesting electronic characteristics.

## 9. Conclusions

The great majority of fullerene organometallic compounds belong to two groups; those with η^2^ and those with η^5^ hapticities, however some examples of this type of compound manifest the other four possible hapticities; thus the field is open and there are several opportunities for working on theoretical [62] as well as experimental [63] projects for the purpose of studying these structures, their applications and reaching an understanding of both bond and reactivity trends.
